# In distributive phosphorylation catalytic constants enable non-trivial dynamics

**DOI:** 10.1007/s00285-024-02114-8

**Published:** 2024-06-25

**Authors:** Carsten Conradi, Maya Mincheva

**Affiliations:** 1 Hochschule für Technik und Wirtschaft, Berlin, Germany; 2 Department of Mathematical Sciences, Northern Illinois University, DeKalb, IL USA

**Keywords:** Distributive phosphorylation, Extreme vector, Hopf bifurcation, Sustained oscillations

## Abstract

Ordered distributive double phosphorylation is a recurrent motif in intracellular signaling and control. It is either sequential (where the site phosphorylated last is dephosphorylated first) or cyclic (where the site phosphorylated first is dephosphorylated first). Sequential distributive double phosphorylation has been extensively studied and an inequality involving only the catalytic constants of kinase and phosphatase is known to be sufficient for multistationarity. As multistationarity is necessary for bistability it has been argued that these constants enable bistability. Here we show for cyclic distributive double phosphorylation that if its catalytic constants satisfy an analogous inequality, then Hopf bifurcations and hence sustained oscillations can occur. Hence we argue that in distributive double phosphorylation (sequential or distributive) the catalytic constants enable non-trivial dynamics. In fact, if the rate constant values in a network of cyclic distributive double phosphorylation satisfy this inequality, then a network of sequential distributive double phosphorylation with the *same rate constant values* will show multistationarity—albeit for different values of the total concentrations. For cyclic distributive double phosphorylation we further describe a procedure to generate rate constant values where Hopf bifurcations and hence sustained oscillations can occur. This may, for example, allow for an efficient sampling of oscillatory regions in parameter space. Our analysis is greatly simplified by the fact that it is possible to reduce the network of cyclic distributive double phosphorylation to what we call a network with a single extreme ray. We summarize key properties of these networks.

**Supplementary Information:**

The online version contains supplementary material available at 10.1007/s00285-024-02114-8.

## Introduction

Phosphorylation is a process where proteins are altered by adding and removing phosphate groups at designated binding sites. It is a recurrent motif in many large reaction networks involved in intracellular signaling and control (Suwanmajo and Krishnan, 2018). Often spatial effects are neglected and the time dynamics of the participating chemical species concentrations is described by ordinary differential equations. There exists a plethora of small ODE models that include phosphorylation at one or two binding sites together with the interaction of various regulating chemical species, see, for example, Ramesh et al. (2023). These models exhibit a wide range of dynamical properties ranging from multistationarity and bistability to sustained oscillations (Ramesh et al. 2023).

**Fig. 1:**
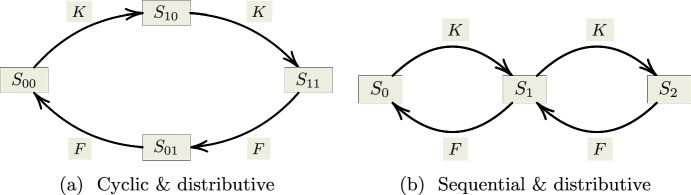
Reaction schemes describing distributive double phosphorylation of a protein *S* at two binding sites by a kinase *K* and a phosphatase *F*. **a** cyclic double phosphorylation, **b** distributive. In **a** the subscript $_{ij}$ denotes the state of the phosphorylation sites: 0 unphosphorylated, 1 phosphorylated (e.g. $S_{10}$ denotes those molecules of *S*, where the first site is phosphorylated and the second site is unphosphorylated). In **b** the subscript $_i$ denotes the number of attached phosphate groups (e.g. $S_0$ denotes unphosphorylated protein)

Phosphorylation and dephosphorylation are catalyzed by two enzymes, a kinase and a phosphatase. As described in Carlos Salazar (2007), Salazar and Höfer (2009), this process can either be processive or distributive: if it is processive, then all available binding sites are phosphorylated or de-phosphorylated upon binding of protein and kinase/phosphatase. If the process is distributive, then at most one binding site is modified upon each binding of protein and kinase or phosphatase. Furthermore, multisite phosphorylation and dephosphorylation can occur at a random sequence of binding sites or at an ordered sequence of binding sites. An ordered mechanism is either sequential or cyclic (Carlos Salazar 2007; Salazar and Höfer 2009). In a sequential mechanism the last site to be phosphorylated is dephosphorylated first, while in a cyclic mechanism the first site to be phosphorylated is also dephosphorylated first—as depicted in the reaction schemes of Fig. 1.

Networks of sequential phosphorylation have been studied extensively and it has been shown that already networks without any form of regulation can exhibit non-trivial dynamics. It is, for example, known that models of sequential and processive phosphorylation and dephosphorylation have a unique, globally attracting steady state (Conradi and Shiu 2015). For sequential distributive phosphorylation and dephosphorylation multistationarity and bistability have been established (Conradi et al. 2008; Hell and Rendall 2015; Holstein et al. 2013). And for a mixed mechanism of sequential distributive phosphorylation and processive dephosphorylation at two binding sites Hopf bifurcations and sustained oscillations have been described in Suwanmajo and Krishnan (2015) and Conradi et al. (2019). For more information about the dynamics of multisite phosphorylation systems see the review (Conradi and Shiu 2018) and the references therein.

Networks of cyclic phosphorylation have been studied in Suwanmajo et al. (2020). The authors study in particular the following mass action network (1) derived from the reaction scheme of Fig. 1a (where the notation is the same as in Fig. 1a and $K S_{00}$, $K S_{10}$, $F S_{11}$ and $F S_{01}$ denote enzyme-substrate complexes):1
Suwanmajo et al. (2020) it is shown that for every admissible positive value of the total concentrations and all positive values of the reaction rate constants there exists a unique positive steady state. The authors furthermore provide parameter values where a Hopf bifurcation occurs (with the total amount of kinase as a bifurcation parameter) and sustained oscillations emerge (Suwanmajo et al. 2020, Fig. 4). The authors however neither describe how the respective parameter values were found nor do they explain how parameter values leading to oscillations can be found.

Here we study the same network (1) and provide an answer to the latter question. In particular, we show that if the rate constants $\kappa _3$, $\kappa _6$, $\kappa _9$ and $\kappa _{12}$ satisfy the inequality

2 $$\begin{aligned} \kappa _3\, \kappa _9 - \kappa _6\, \kappa _{12} <0, \end{aligned}$$

then Hopf-bifurcations and sustained oscillations can occur. In enzyme catalysis these constants are known as *catalytic constants* [cf. the discussion in Sect. 4 or Conradi and Mincheva (2014)].

To be more precise, we analyze the following irreversible subnetwork of (1)3and show that if the values of the catalytic constants $\kappa _3$, $\kappa _6$, $\kappa _9$ and $\kappa _{12}$ satisfy (2), then there exist values for $\kappa _1$, $\kappa _4$, $\kappa _7$, $\kappa _{10}$ and the total concentrations such that the steady state Jacobian of network (3) has a complex-conjugate pair of eigenvalues on the imaginary axis. Furthermore, if this eigenvalue pair crosses the imaginary axis as one of the parameters is varied, then a Hopf bifurcation occurs at this particular steady state (Theorem 3.4). We describe a derivative condition for this crossing and a procedure to find such parameter values. Based on a result by Banaji (2018), we then argue that if a supercritical Hopf bifurcation occurs in network (3) and a stable limit cycle emerges, then the full network (1) will have a stable limit cycle as well (for appropriately chosen values of $\kappa _2$, $\kappa _5$, $\kappa _8$ and $\kappa _{11}$). This is Theorem 3.9.

Finally, we compare our results for *cyclic* distributive double phosphorylation with those obtained in Conradi and Mincheva (2014) for *sequential* distributive double phosphorylation. In Conradi and Mincheva (2014) an inequality that is sufficient for multistationarity has been described. Remarkably this inequality involves the catalytic constants of sequential distributive double phosphorylation in the same way as our inequality (2) involves the catalytic constants of cyclic distributive double phosphorylation. As multistationarity is necessary for bistability one may argue that these rate constants enable bistability in sequential and distributive double phosphorylation. Hence we conclude that in distributive phosphorylation (sequential or cyclic) these constants enable non-trivial dynamics.

The paper is organized as follows: to arrive at our results the ODEs derived from network (1) and (3) are analyzed. For this purpose we recall in Sect. 2 some well known facts about ODEs defined by reaction networks and introduce a special class of reaction networks that we call networks with a single extreme ray. In this section we also derive conditions for a simple Hopf bifurcation in such networks. In Sect. 3 we first analyze network (3) and verify these bifurcation conditions in Theorem 3.4. Then we turn to the full network (1) and Theorem 3.9. We also present the procedure to determine rate constant and total concentration values. In Sect. 4 we discuss inequality (2) in the light of the the results presented in Conradi and Mincheva (2014). Appendix A and B contain some of the longer proofs of the results presented in Sect. 2. Appendix C–E contain information to reproduce the numerical results displayed in the figures throughout the paper.

## Biochemical reaction networks with mass action kinetics

To establish our results we exploit the special structure of the Jacobian of a certain class of reaction networks that we call *networks with a single extreme ray*. We introduce this class here in full generality. To this end we first consider a general reaction network with *n* species and *r* reactions in Sect. 2.1 and recall the structure of the ODEs defined by such a general network. In Sect. 2.2 we discuss steady states and formally define networks with a single extreme ray. In Sect. 2.3 we present a formula for the Jacobian of a general reaction network at steady state. In Sect. 2.4 we discuss the steady state Jacobian of networks with a single extreme ray. Finally, in Sect. 2.5 we present conditions for simple Hopf bifurcations in networks of this kind.

### Reaction networks with *n* species and *r* reactions

We briefly introduce the relevant notation, for a more detailed discussion we refer to the large body of literature on mass action networks, for example, Conradi and Flockerzi (2012) or Conradi and Pantea (2019).

To every chemical species we associate a variable $x_i$ denoting its concentration. For network (1) and (3) we use the association as given in Table 1.

**Table 1:** Species and concentration variables for network (1) and (3)

$x_1$	$x_2$	$x_3$	$x_4$	$x_5$	$x_6$	$x_7$	$x_8$	$x_9$	$x_{10}$
*K*	*F*	$$S_{00}$$	$$S_{10}$$	$$S_{01}$$	$$S_{11}$$	$$K S_{00} $$	$$KS_{10}$$	$$FS_{01} $$	$$FS_{11} $$

Consider network (1), nodes like $S_{00}+K$ are called complexes. To every complex we associate a vector $y\in {\mathbb {R}}^n$ representing the stoichiometry of the associated chemical species. The complex $S_{00}+K$ consists of one unit of $S_{00}$ and one unit of *K*, hence, in the ordering of Table 1 the vector $y^T=(1,0,1,0,0,0,0,0,0,0)$ represents its stoichiometry. In a similar way one arrives at the ten complex vectors for networks (1) and (3) given in Table 3 of Appendix C.

To every reaction $r^{(l)}$ we associate the difference of the complexes at the tip and the tail of the reaction arrow. For example, to the reaction $S_{00}+K \rightarrow S_{00}K$ we associate the vector $r^{(l)}=y^{(2)}-y^{(1)} = (-1,-1,0,1,0,0,0,0,0,0)^T$ (using the labeling of complexes given in Table 3 of Appendix C). All reaction vectors $r^{(i)}$ are collected as columns of the stoichiometric matrix $S\in {\mathbb {R}}^{n\times r}$:

4 $$\begin{aligned} S=\left[ \begin{array}{ccc} r^{(1)}&\dots&r^{(r)} \end{array} \right] . \end{aligned}$$

To every reaction $r^{(l)}$ we further associate a reaction rate function $v_l(k,x)$ describing the ‘speed’ of the reaction. We consider only mass action kinetics, hence the reaction rate function of reaction $r^{(l)}: y^{(i)}\rightarrow y^{(j)}$ is given by the monomial function

$$\begin{aligned} v_l(k,x) = k_l\, \prod _{l=1}^n x_l^{y_l^{(i)}} = k_l\, x^{y^{(i)}}, \end{aligned}$$

where $k_l$ is a parameter called the rate constant and $x^y$ is the customary shorthand notation for the product $\prod _{l=1}^n x_l^{y_l}$ of two *n*-vectors *x* and *y*.

We collect the vectors at the tail of every reaction as columns of a matrix *Y* and note that this matrix may contain several copies of the same vector:

5 $$\begin{aligned} Y=\left[ \begin{array}{ccc} y^{(1)}&\dots&y^{(r)} \end{array} \right] \end{aligned}$$

We collect all rate constants in a vector $$k^T=(k_1$$, ..., $$k_r)$$ and the monomials $$x^{y^{(i)}}$$ in a vector

6 $$\begin{aligned} \phi (x) = (x^{y^{(1)}}, \ldots , x^{y^{(r)}})^T\, \end{aligned}$$

where the vectors $y^{(i)}$ reference the columns of the matrix *Y* defined in  (5). The reaction rate function is then defined using *k* and $\phi (x)$ as

7 $$\begin{aligned} v(k,x) = {{\,\textrm{diag}\,}}(k)\, \phi (x). \end{aligned}$$

After an ordering of species and reactions is fixed, every reaction network with mass action kinetics defines a matrix *S* and a reaction rate function *v*(*k*, *x*) in a unique way. These objects in turn define the following system of ODEs:

8 $$\begin{aligned} \dot{x} = S v(k,x)\, \end{aligned}$$

where $\dot{x}$ denotes the vector of derivatives with respect to time. Example are abundant in the literature, see, for example, Conradi and Flockerzi (2012, Sect. 2) or Conradi and Pantea (2019).

Often the matrix *S* does not have full row rank. In this case let *W* be a matrix whose columns span $\ker (S^T)$, that is a full rank matrix *W* with $W^T\, S \equiv 0$. Then $W^T\, \dot{x} \equiv 0$ and for every solution *x*(*t*) with initial value $x(0)=x_0$ one obtains

$$\begin{aligned} W^T\, x(t) = W^T\, x_0 = \text {const. } \end{aligned}$$

Hence, if ${{\,\textrm{rank}\,}}(S)=s<n$, then one obtains $n-s$ conservation relations

9 $$\begin{aligned} W^T\, x = c. \end{aligned}$$

### Steady states

We are interested in points (*k*,*x*) that are solutions of

10 $$\begin{aligned} S v(k,x) = 0. \end{aligned}$$

If (*k*,*x*) is a solution of (10), then *x* is a steady state of (8) for the rate constants *k*.

We proceed as in Conradi et al. (2020) and express the reaction rates *v*(*k*, *x*) at a steady state as a nonnegative combination of the extreme vectors of the pointed polyhedral cone $\ker (S)\cap {\mathbb {R}}_{\ge 0}^r$. This idea goes back to Clarke and coworkers (cf. for example, Clarke 1988; Conradi and Shiu 2018) and it is as follows: (*k*,*x*) satisfy (10), if and only if the corresponding *v*(*k*, *x*) is such that $v(k,x)\in \ker (S)\cap {\mathbb {R}}_{\ge 0}^r$ (cf. e.g. Conradi et al. 2020).

Convex polyhedral cones have a finite number of extreme vectors (up to a scalar positive multiplication Rockafellar 1970). Therefore, any element *v* of such a cone can be represented as a nonnegative linear combination of its extreme vectors $\{E_1, \ldots ,E_l \}$

11 $$\begin{aligned} v = \sum _{i=1}^l \lambda _i E_i = E \lambda ,\; \lambda \in {\mathbb {R}}_{\ge 0}^l, \end{aligned}$$

where *E* is the matrix with columns $E_1,\dots ,E_l$ and $\lambda ^T=(\lambda _1,\dots ,\lambda _l)$.

#### Remark 2.1

(*The relative interior of* $\ker (S)\cap {\mathbb {R}}_{\ge 0}^r$)

(A) As explained in, for example, Telek and Feliu (2023), a system (8) has *positive* solutions (*k*,*x*), if and only if the matrix *E* does not contain a zero row. Hence we will only consider networks where the matrix *E* are of this kind.
(B) We are only interested in positive values of *k* and *x*. Thus we are only interested in *v*(*k*, *x*) that are strictly positive and hence belong to the *relative interior* of the cone $\ker (S)\cap {\mathbb {R}}_{\ge 0}^r$. Consequently we are only interested in those $\lambda \in {\mathbb {R}}_{\ge 0}^l$ that yield a positive $E\lambda$. In the remainder of the paper we therefore restrict $\lambda \in {\mathbb {R}}_{\ge 0}^l$ to the set:
  12 $$\begin{aligned} \Lambda (E):= \left\{ \lambda \in {\mathbb {R}}_{\ge 0}^l | E\lambda >0 \right\} . \end{aligned}$$
  This set has been introduced in Conradi and Flockerzi (2012), cf. Conradi and Flockerzi (2012, Remark 4).
(C) A more detailed discussion of the relation between positive solutions of (10), the cone $\ker (S)\cap {\mathbb {R}}_{\ge 0}^r$ and the generator matrix *E* can be found in Telek and Feliu (2023), cf. in particular (Telek and Feliu 2023, Proposition 6).

In the analysis of network (1) we will later analyze subnetworks that fit the following definition:

#### Definition 1

(*Networks with a single extreme ray*) Consider a reaction network with stoichiometric matrix *S*. If the cone $\ker (S)\cap {\mathbb {R}}_{\ge 0}^r$ is generated by a single positive vector then we call it a *network with a single extreme ray*.

As discussed in Remark 2.1, if $x>0$ and $k>0$, then $v(k,x)={{\,\textrm{diag}\,}}(k)\phi (x)>0$. Consequently, if (*k*,*x*) satisfy the steady state Eq. (10), then $v(k,x)\in \ker (S)\cap {\mathbb {R}}_{\ge 0}^r$ and there exists a vector $\lambda \in \Lambda \left( E\right)$, such that (11) is satisfied. Thus one may parameterize all reaction rates at steady states via the equation

13 $$\begin{aligned} {{\,\textrm{diag}\,}}(k)\, \phi (x) = E\, \lambda ,\; \lambda \in \Lambda \left( E\right) . \end{aligned}$$

Likewise, given some $\lambda \in \Lambda \left( E\right)$ and a positive *x* one obtains a positive vector *k* such that (*k*,*x*) satisfy the steady state Eq. (10) by the following formula:

14 $$\begin{aligned} k = {{\,\textrm{diag}\,}}(\phi (h))E\lambda , \end{aligned}$$

where

15 $$\begin{aligned} h=\frac{1}{x} \;. \end{aligned}$$

We observe that (i) the formula (15) is well defined as by assumption $x>0$, (ii) that the vector *k* from (14) is positive since all entries of $E\lambda$ are positive (as, by assumption, the matrix *E* does not have any zero rows, cf. Remark 2.1) and (iii) that the formula (14) is obtained by solving the *k*-linear Eq. (13) for *k*.

### The Jacobian at steady state

Equations (11) and (13) introduce a parametrization of the reaction rates *v*(*k*, *x*) at steady states in terms of the convex parameters *h* and $\lambda$. This parametrization can be used to parameterize the Jacobian at steady state: as explained in detail in Conradi et al. (2020) or Clarke (1988), if (*k*,*x*) satisfy the steady state Eq. (10) and hence *v*(*k*, *x*) is such that (13) holds, then the Jacobian evaluated at that (*k*, *x*) is given by the following formula (cf. Conradi et al. 2020, Proposition 2):

16 $$\begin{aligned} J(k,x) = J(h,\lambda ) = S {{\,\textrm{diag}\,}}(E\lambda ) Y^T {{\,\textrm{diag}\,}}(h), \end{aligned}$$

where *Y* denotes the matrix introduced in (5).

### The Jacobian of networks with a single extreme vector

For any reaction network where the cone $\ker (S)\cap {\mathbb {R}}_{\ge 0}^r$ is spanned by a single positive vector *E* the parameter $\lambda$ is a positive scalar and formula (16) is equivalent to

17 $$\begin{aligned} J_{\lambda }(h) = \lambda S {{\,\textrm{diag}\,}}(E)Y^T {{\,\textrm{diag}\,}}(h). \end{aligned}$$

We use $J_{\lambda }(h)$ to denote the Jacobian in this special case.

Here we comment on the characteristic polynomial of general matrices $J_{\lambda }(h)$, that is on the polynomial $\det (\mu I - J_{\lambda }(h))$ of an $n\times n$ Jacobian of the form (17). We will assume that ${{\,\textrm{rank}\,}}(J_{\lambda }) =s \le n$ and we will use the symbol $a_i(h,\lambda )$ to denote the coefficients of its characteristic polynomial. We will also discuss the case $\lambda =1$, that is, the characteristic polynomial $\det (\mu I - J_1(h))$ of $J_1(h)$ with coefficients $b_i(h)$. For this purpose, the characteristic polynomial of the Jacobian $J_{\lambda } (h)$ is denoted by

18 $$\begin{aligned} \det (\mu I -J_{\lambda } (h)) = \mu ^{n-s}(\mu ^s+ a_1 (\lambda , h) \mu ^{s-1} + \cdots + a_s(\lambda ,h)), \end{aligned}$$

while the characteristic polynomial of $J_{1} (h)$ is denoted by

19 $$\begin{aligned} \det (\mu I -J_{1} (h)) = \mu ^{n-s}(\mu ^s+ b_1 (h) \mu ^{s-1} + \cdots + b_s (h)) \;. \end{aligned}$$

Concerning these polynomials we have the following corollary of Lemma A.4 in Appendix A where we show the relationship between the coefficients $a_i(h,\lambda )$ and $b_i (h)$.

#### Corollary 2.2

Suppose the matrix *E* consists of a single positive column vector. Let $J_{\lambda }(h)$ be as in (17) with ${{\,\textrm{rank}\,}}(J_{\lambda }(h))={{\,\textrm{rank}\,}}(J_1(h)) =s <n$ and let $a_i(\lambda ,h)$, $b_i(h)$ be the coefficients of the characteristic polynomials in (18) and in (19). Then the coefficients $a_i(\lambda ,h)$ and $b_i(h)$ satisfy:

20 $$\begin{aligned} a_i(\lambda ,h) = \lambda ^i b_i(h),\, i=1,\, \ldots ,\, s. \end{aligned}$$

Moreover, the polynomial $\det (\mu I - J_{\lambda }(h))$ is given by the following formula:

21 $$\begin{aligned} \det (\mu I_n - J_{\lambda } (h))= & {} \det (\mu I_n-\lambda J_1 (h)) \nonumber \\= & {} \mu ^{n-s} \lambda ^s \left( \left( \frac{\mu }{\lambda }\right) ^s + \sum _{i=1}^s b_i(h) \left( \frac{\mu }{\lambda }\right) ^{s-i} \right) \end{aligned}$$

We observe the following consequences of Corollary 2.2:

#### Remark 2.3

(I) By (21) it follows that if $\omega (h)$ is an eigenvalue of $J_1(h)$, then $\mu (\lambda ,\omega ) = \lambda \omega (h)$ is an eigenvalue of $J_{\lambda }(h)$.
(II) In our setting $\lambda >0$, hence the sign of the real part of the eigenvalues $Re(\mu (\lambda ,h))$ of $J_{\lambda }(h)$ is independent of $\lambda$: ${{\,\textrm{sign}\,}}(Re(\mu (\lambda ,h))) = {{\,\textrm{sign}\,}}(Re(\omega (h)))$.
(III) In particular, the matrix $J_{\lambda }(h)$ has a purely imaginary pair of eigenvalues $\pm i \lambda \omega (h)$ if and only if $J_1 (h)$ has a purely imaginary pair $\pm i \omega (h)$.

### Simple Hopf bifurcations in networks with a single extreme ray

We recall the definition of a *simple Hopf bifurcation* for a parameter dependent system of ODEs of the form ${\dot{x}} = g_p(x)$, where $x \in {\mathbb {R}}^s$, and $g_p(x)$ varies smoothly in *p* and *x*. Let $x^* \in {\mathbb {R}}^s$ be a steady state of the ODE system for some fixed value $p_0$, that is, $g_{p_0}(x^*)=0$. Furthermore, we assume that we have a smooth curve of steady states around $p_0$:

$$\begin{aligned} p ~\mapsto ~ x(p)~ \text {with }x(p_0)=x^*. \end{aligned}$$

That is, $g_{p}\left( x(p) \right) = 0$ for all *p* close enough to $p_0$.

Further let *J*(*x*(*p*), *p*) be the Jacobian of $g_p(x)$ evaluated at *x*(*p*). If, as *p* varies, a complex-conjugate pair of eigenvalues of *J*(*x*(*p*), *p*) crosses the imaginary axis, then there exists a Hopf bifurcation at $(x(p_0,p_0))$. A *simple Hopf bifurcation* occurs at $(x(p_0),p_0)$, if no other eigenvalue crosses the imaginary axis at the same value $p_0$. In this case, a limit cycle arises. If the Hopf bifurcation is supercritical, then stable periodic solutions are generated for nearby parameter values (Liu 1994).

Similar to Conradi et al. (2019) and Conradi et al. (2020) we want to build on results described in Liu (1994) and Yang (2002) to establish Hopf bifurcations. As in previous work (cf. e.g. Conradi et al. 2020; Liu 1994; Yang 2002), we will use a criterion based on the following Hurwitz determinants:

#### Definition 2

The *i*-*th Hurwitz matrix* of a univariate polynomial $p(z)= a_0 z^s + a_{1} z^{s-1} + \cdots + a_s$ is the following $i \times i$ matrix:

$$\begin{aligned} H_i = \begin{pmatrix} a_1 &{} a_0 &{} 0 &{} 0 &{} 0 &{} \cdots &{} 0 \\ a_3 &{} a_2 &{} a_1 &{} a_0 &{} 0 &{} \cdots &{} 0 \\ \vdots &{} \vdots &{} \vdots &{}\vdots &{} \vdots &{} &{} \vdots \\ a_{2i-1} &{} a_{2i-2} &{} a_{2i-3} &{} a_{2i-4} &{}a_{2i-5} &{}\cdots &{} a_i \end{pmatrix}~, \end{aligned}$$

in which the (*k*, *l*)-th entry is $a_{2k-l}$ as long as $0\le 2 k - l \le s$, and 0 otherwise. The determinants $\det (H_i)$ are called *Hurwitz determinants*.

To every square matrix one can associate Hurwitz matrices via its characteristic polynomial in an analogous way. In the following we consider two families of Hurwitz matrices constructed according to Definition 2: matrices $H_l(\lambda ,h)$ obtained from coefficients $a_i(\lambda ,h)$ of (18) and matrices $G_l(h)$ of coefficients $b_i(h)$ of (19). Our analysis is greatly simplified by the relationship between the two families established in Proposition 2.4 below (a proof is given in Appendix B):

#### Proposition 2.4

The Hurwitz determinants for the characteristic polynomials (18) and (19) satisfy the following equation

$$\begin{aligned} \det \left( H_{l} (\lambda , h)\right) = \lambda ^{l(l+1)/2} \det \left( G_{l} (h)\right) , \quad l=1,2, \ldots ,s. \end{aligned}$$

The following proposition is a specialization of Conradi et al. (2020, Proposition 1) and Yang (2002, Theorem 2) to a network where $\ker (S)\cap {\mathbb {R}}_{\ge 0}^r$ is generated by a single positive vector. It implies that for such networks to detect a simple Hopf bifurcation one only needs to study the polynomial (19) and its Hurwitz matrices $G_i$, $i=1,2,\ldots , s$.

#### Proposition 2.5

Let ${\dot{x}} = S v(k,x)$ be an ODE system model for a reaction network with mass action kinetics where ${{\,\textrm{rank}\,}}(S)=s$. Suppose that the matrix *E* in (11) consists of a single positive vector and let $J_{\lambda }( h)$ be the corresponding Jacobian in convex parameters. Further let the characteristic polynomials of $J_{\lambda } (h)$ and $J_{1} (h)$ be as in (18) and (19), respectively. If there exists a fixed value $h=h^*$ such that

22 $$\begin{aligned}{} & {} b_s(h^*)>0\quad \textrm{and} \nonumber \\{} & {} \det (G_1(h^*))> 0, \ldots , \det (G_{s-2}(h^*)) > 0\quad \textrm{and} \nonumber \\{} & {} \det (G_{s-1}(h^*))= 0, \end{aligned}$$

then

- $J_{1 } (h^*)$ has a single pair of purely imaginary eigenvalues,
- $J_{\lambda } (h^*)$ has a single pair of purely imaginary eigenvalues for all $\lambda >0$,
- for the dynamical system ${\dot{x}}= S v(k,x)$ there exists a simple Hopf bifurcation at $h=h^*$ for all $\lambda >0$ if there exists some $l \in \{1,2, \ldots , n \}$ such that
  23 $$\begin{aligned} \frac{\partial \det (G_{n-1})}{\partial h_l}\Bigg |_{h_l=h_l^*} \ne 0. \end{aligned}$$

#### Proof

- The proof follows by Conradi et al. (2020, Proposition 1).
- By Corollary 2.2${{\,\textrm{sign}\,}}(b_s(h^*))={{\,\textrm{sign}\,}}(a_s(h^*,\lambda ))$ for all $\lambda >0$. Likewise, by Proposition 2.4${{\,\textrm{sign}\,}}(\det (G_l(h^*)))={{\,\textrm{sign}\,}}(\det (H_l(h^*,\lambda )))$, $l=1$, ..., *s* for all $\lambda >0$. Hence by (a) and by Conradi et al. (2020, Proposition 1) the Jacobian $J_{\lambda } (h^*)$ has a single pair of purely imaginary eigenvalues for all $\lambda >0$.
- The fact that $J_{\lambda } (h_l^*)$ for all $\lambda >0$ has a single pair of purely imaginary eigenvalues has been established in (b). Proposition 2.4 implies the following relationship between the two derivatives of $\det \left( H_{s-1}(h,\lambda )\right)$ and $\det \left( G_{s-1}(h,\lambda ) \right)$ with respect to the $h_l$:
  $$\begin{aligned} \frac{\partial \det (H_{s-1}(h,\lambda ))}{\partial h_l} = \lambda ^{\frac{s(s+1)}{2}} \frac{\partial \det (G_{s-1}(h))}{\partial h_l},\quad l=1, \ldots , n. \end{aligned}$$
  Thus, if (23) holds for some $l\in \{ 1$, ..., $n\}$, then, for the same *l*, one has
  $$\begin{aligned} \frac{\partial \det (H_{s-1}(h,\lambda ))}{\partial h_l}\Bigg |_{(h^*,\lambda )} \ne 0 \text { for all }\lambda >0. \end{aligned}$$
  Therefore, by (a), (b) and by Yang (2002, Theorem 2, Remark 2), (c) follows as well.

$$\square $$

#### Remark 2.6

The parameter $\lambda$ is not suited as a bifurcation parameter in (23) as by Proposition 2.4$\det (H_{s-1}(h,\lambda )) = \lambda ^{\frac{s(s-1)}{2}} \det (G_{s-1}(h))$. Hence $\frac{\partial \det (H_{s-1}(h,\lambda ))}{\partial \lambda }|_{h=h^*} = 0$, whenever $\det (G_{s-1}(h^*))=0$ (cf. Remark 2.3 explaining that the existence of a pair of purely imaginary eigenvalues is independent of $\lambda$).

## Analysis of networks of cyclic distributive double phosphorylation

To derive a dynamical model of network (1) we assign the variables given in Table 1 to the chemical species and obtain the following ODEs:

24a $$\begin{aligned} \dot{x}_{1}&= -\kappa _{1} x_{1} x_{3}- \kappa _{4} x_{1} x_{4}+ (\kappa _{2} +\kappa _{3}) x_{7}+ (\kappa _{5} +\kappa _{6}) x_{8} \end{aligned}$$

24b $$\begin{aligned} \dot{x}_{2}&= -\kappa _{10} x_{2} x_{5}-\kappa _{7} x_{2} x_{6}+ (\kappa _{8} +\kappa _{9}) x_{10} +(\kappa _{11}+\kappa _{12}) x_{9} \end{aligned}$$

24c $$\begin{aligned} \dot{x}_{3}&= -\kappa _{1} x_{1} x_{3}+ \kappa _{2} x_{7}+\kappa _{12} x_{9} \end{aligned}$$

24d $$\begin{aligned} \dot{x}_{4}&= -\kappa _{4} x_{1} x_{4}+\kappa _{3} x_{7}+\kappa _{5} x_{8} \end{aligned}$$

24e $$\begin{aligned} \dot{x}_{5}&= -\kappa _{10} x_{2} x_{5} + \kappa _{9} x_{10}+\kappa _{11} x_{9} \end{aligned}$$

24f $$\begin{aligned} \dot{x}_{6}&= -\kappa _{7} x_{2} x_{6} + \kappa _{8} x_{10}+\kappa _{6} x_{8} \end{aligned}$$

24g $$\begin{aligned} \dot{x}_{7}&= -(\kappa _{2} +\kappa _{3}) x_{7} + \kappa _{1} x_{1} x_{3} \end{aligned}$$

24h $$\begin{aligned} \dot{x}_{8}&= -(\kappa _{5} +\kappa _{6}) x_{8} + \kappa _{4} x_{1} x_{4}\end{aligned}$$

24i $$\begin{aligned} \dot{x}_{9}&= -(\kappa _{11} +\kappa _{12}) x_{9} + \kappa _{10} x_{2} x_{5} \end{aligned}$$

24j $$\begin{aligned} \dot{x}_{10}&= -(\kappa _{8} +\kappa _{9} ) x_{10}+\kappa _{7} x_{2} x_{6} \end{aligned}$$

Using the same variables $x_1$, ..., $x_{10}$ as in Table 1, we derive the following ODEs from network (3):

25a $$\begin{aligned} \dot{x}_{1}&= -\kappa _1 x_{1} x_{3}-\kappa _4 x_{1} x_{4}+\kappa _3 x_{7}+\kappa _6 x_{8}, \end{aligned}$$

25b $$\begin{aligned} \dot{x}_{2}&= \kappa _9 x_{10}-\kappa _{10} x_{2} x_{5}-\kappa _7 x_{2} x_{6}+\kappa _{12} x_{9}, \end{aligned}$$

25c $$\begin{aligned} \dot{x}_{3}&= -\kappa _1 x_{1} x_{3}+\kappa _{12} x_{9}, \end{aligned}$$

25d $$\begin{aligned} \dot{x}_{4}&= -\kappa _4 x_{1} x_{4}+\kappa _3 x_{7}, \end{aligned}$$

25e $$\begin{aligned} \dot{x}_{5}&= \kappa _9 x_{10}-\kappa _{10} x_{2} x_{5}, \end{aligned}$$

25f $$\begin{aligned} \dot{x}_{6}&= -\kappa _7 x_{2} x_{6}+\kappa _6 x_{8}, \end{aligned}$$

25g $$\begin{aligned} \dot{x}_{7}&= \kappa _1 x_{1} x_{3}-\kappa _3 x_{7}, \end{aligned}$$

25h $$\begin{aligned} \dot{x}_{8}&= \kappa _4 x_{1} x_{4}-\kappa _6 x_{8}, \end{aligned}$$

25i $$\begin{aligned} \dot{x}_{9}&= \kappa _{10} x_{2} x_{5}-\kappa _{12} x_{9}, \end{aligned}$$

25j $$\begin{aligned} \dot{x}_{10}&= -\kappa _9 x_{10}+\kappa _7 x_{2} x_{6}. \end{aligned}$$

Both networks have the same set of three conservation relations, one for the total amount of kinase ($c_1$), phosphatase ($c_2$), and substrate ($c_3$), respectively:

26 $$\begin{aligned}&x_{1}+x_{7}+x_{8} = c_1 \nonumber \\&x_{2}+x_{9}+ x_{10} = c_2 \nonumber \\&x_{3}+x_{4}+x_{5}+x_{6}+x_{7}+x_{8}+x_{9}+x_{10} = c_3 \end{aligned}$$

### Steady states of network (3)

For the network (3) the matrix *E* consists of a single vector of all 1:

27 $$\begin{aligned} E^T=(1,1,1,1,1,1,1,1), \end{aligned}$$

hence network (3) is a network with a single extreme ray and we can apply the results of Sects. 2.4 and 2.5.

Given *E* from (27) condition (13) consequently becomes (cf. Eq. (49) of Appendix C for *v*(*k*, *x*)):

$$\begin{aligned} \kappa _1 x_{1} x_{3} = \kappa _3 x_{7} = \kappa _4 x_{1} x_{4} = \kappa _6 x_{8} = \kappa _7 x_{2} x_{6} = \kappa _9 x_{10} = \kappa _{10} x_{2} x_{5} = \kappa _{12} x_{9} = \lambda . \end{aligned}$$

These equations can be solved for *x* (in terms of *k* and $\lambda$) to obtain the following steady state parametrization:

28 $$\begin{aligned}{} & {} x_{3} = \frac{\lambda }{\kappa _1 x_{1}},\quad x_{4} = \frac{\lambda }{\kappa _4 x_{1}},\quad x_{5} = \frac{\lambda }{\kappa _{10} x_{2}},\quad x_{6} = \frac{\lambda }{\kappa _7 x_{2}}, \nonumber \\{} & {} x_{7} = \frac{\lambda }{\kappa _3},\quad x_{8} = \frac{\lambda }{\kappa _6},\, x_{9} = \frac{\lambda }{\kappa _{12}},\quad x_{10} = \frac{\lambda }{\kappa _9}, \end{aligned}$$

where $x_1$ and $x_2$ are arbitrary positive numbers. Similarly, solving for *k* one obtains

29 $$\begin{aligned}{} & {} \kappa _1 = h_1 h_3\lambda , \quad \kappa _3 = h_7 \lambda , \quad \kappa _4 = h_1 h_4\lambda , \quad \kappa _6 = h_8 \lambda , \nonumber \\{} & {} \kappa _7 = h_2 h_6\lambda ,\quad \kappa _9 = h_{10} \lambda , \quad \kappa _{10} = h_2 h_5 \lambda , \quad \kappa _{12} = h_9\lambda , \end{aligned}$$

where $h_i=\frac{1}{x_i}$, $i=1$, ..., 10 (this is (14) for network (3) with *v*(*k*, *x*) given in Appendix C).

### Simple Hopf bifurcations for network (3)

The Jacobian $J_{\lambda } (h)$ of network (3) computed via (16) is

30 $$\begin{aligned} \tiny J_{\lambda }(h) = \lambda \, \left[ \begin{array}{rrrrrrrrrr} -2 h_{1} &{} 0 &{} -h_{3} &{} -h_{4} &{} 0 &{} 0 &{} h_{7} &{} h_{8} &{} 0 &{} 0 \\ 0 &{} -2 h_{2} &{} 0 &{} 0 &{} -h_{5} &{} -h_{6} &{} 0 &{} 0 &{} h_{9} &{} h_{10} \\ -h_{1} &{} 0 &{} -h_{3} &{} 0 &{} 0 &{} 0 &{} 0 &{} 0 &{} h_{9} &{} 0 \\ -h_{1} &{} 0 &{} 0 &{} -h_{4} &{} 0 &{} 0 &{} h_{7} &{} 0 &{} 0 &{} 0 \\ 0 &{} -h_{2} &{} 0 &{} 0 &{} -h_{5} &{} 0 &{} 0 &{} 0 &{} 0 &{} h_{10} \\ 0 &{} -h_{2} &{} 0 &{} 0 &{} 0 &{} -h_{6} &{} 0 &{} h_{8} &{} 0 &{} 0 \\ h_{1} &{} 0 &{} h_{3} &{} 0 &{} 0 &{} 0 &{} -h_{7} &{} 0 &{} 0 &{} 0 \\ h_{1} &{} 0 &{} 0 &{} h_{4} &{} 0 &{} 0 &{} 0 &{} -h_{8} &{} 0 &{} 0 \\ 0 &{} h_{2} &{} 0 &{} 0 &{} h_{5} &{} 0 &{} 0 &{} 0 &{} -h_{9} &{} 0 \\ 0 &{} h_{2} &{} 0 &{} 0 &{} 0 &{} h_{6} &{} 0 &{} 0 &{} 0 &{} -h_{10} \\ \end{array} \right] . \end{aligned}$$

For $J_{\lambda }(h)$ given in (30) one has ${{\,\textrm{rank}\,}}(J_{\lambda }(h))=7$. Hence its characteristic polynomial is of the following form (cf. supplementary file S1Cyc_dd_2_coeffs_charpoly.nb and Corollary 2.2):

31 $$\begin{aligned} \det \left( \mu I - J_{\lambda }(h) \right) = \mu ^3 \left( \mu ^7 + \lambda b_1(h) \mu ^{6} + \cdots + \lambda ^{6} b_{6}(h) \mu + \lambda ^7 b_7(h) \right) , \end{aligned}$$

where the coefficients $b_1(h)$, ..., $b_7(h)$ are the coefficients of the characteristic polynomial of the matrix $J_1(h)$. With the next corollary we adapt Proposition 2.5 to the Jacobian of network (3). This allows us to work with the simpler coefficients $b_i(h)$.

#### Corollary 3.1

Consider the dynamical system (25a)–(25j) defined by network (3) with Jacobian $J_{\lambda } (h)$ as in (30). Consider the polynomial (31) for $\lambda =1$ and obtain its coefficients $b_1(h), \ldots ,$ $b_7(h)$ and Hurwitz-matrices $G_1(h)$, ..., $G_6(h)$. Assume that at some $h=h^*$ the following conditions hold:

32 $$\begin{aligned}{} & {} b_7(h^*)>0\quad \textrm{and} \nonumber \\{} & {} \det (G_1(h^*))> 0,\; \ldots ,\;\det (G_5(h^*)) > 0\quad \textrm{and} \nonumber \\{} & {} \det (G_6(h^*))= 0. \end{aligned}$$

Then, for all $\lambda >0$, the dynamical system (25a)–(25j) has a simple Hopf bifurcation at $h=h^*$, if additionally

33 $$\begin{aligned} \exists l\in \{1, \ldots , 10\} \text { such that } \frac{\partial \det (G_6)}{\partial h_l}\Bigg |_{h=h^*} \ne 0. \end{aligned}$$

The coefficients $b_0 (h)$, ..., $b_5 (h)$ and $b_7 (h)$, as well as the Hurwitz determinants $\det (G_2 (h))$, ..., $\det (G_5 (h))$ contain only monomials with positive sign (cf. supplementary file Cyc_dd_2_coeffs_charpoly.nb). This establishes the following Lemma and Corollary:

#### Lemma 3.2

Consider the coefficients of the characteristic polynomial (31) of $J_{\lambda }(h)$ given in (30) for $\lambda =1$ and its Hurwitz determinants. For all $h>0$ the following holds:

(A) $$b_0(h) >0$$, ..., $$b_5(h)>0$$ and $$b_7(h)>0$$ and
(B) $$\det (G_2 (h))>0$$, ..., $$\det (G_5 (h))>0$$.

The Hurwitz determinant $\det (G_6(h))$ contains monomials of both signs (cf. supplementary file cyc_dd_2_detH6.txt), hence it can potentially be zero. To establish this, we consider $\det (G_6(h))$ as a polynomial in $h_1$, $h_2$, $h_3$ and $h_6$ only and study its Newton polytope. Using polymake (Assarf et al. 2017; Gawrilow and Joswig 2000) we compute the following hyperplane representation of this Newton polytope:

$$\begin{aligned} \begin{array}{lll} -h_1 \ge -6 &{}\quad -h_1 - h_2 \ge -11 &{}\quad -h_1 - h_2 - h_3 \ge -15 \\ -h_1 - h_2 - h_3 - h_6 \ge -18 &{}\quad h_6 \ge 0 &{}\quad -h_2 \ge -6 \\ -h_1 - h_3 \ge -11 &{}\quad -h_3 - h_6 \ge -11 &{}\quad -h_2 - h_3 - h_6 \ge -15 \\ -h_1 - h_3 - h_6 \ge -15 &{}\quad h_1 \ge 0 &{}\quad -h_2 - h_3 \ge -11\\ -h_1 - h_2 - h_6 \ge -15 &{}\quad -h_3 \ge -6 &{}\quad -h_2 - h_6 \ge -11 \\ h_2 \ge 0 &{}\quad -h_1 - h_6 \ge -11 &{}\quad -h_6 \ge -6 \\ h_3 \ge 0 \end{array} \end{aligned}$$

We study the coefficients of $\det (G_6)$ as a polynomial in $h_1$, $h_2$, $h_3$ and $h_6$ and find that some of these contain the factor

$$\begin{aligned} h_{10} h_{7}-h_{8} h_{9}. \end{aligned}$$

In fact, visual inspection of all coefficients shows that all monomials with exponent vectors contained in the hyperplane

$$\begin{aligned} h_1 + h_2 + h_3 +h_6 = 18 \end{aligned}$$

have such a coefficient that factors $h_{10} h_{7}-h_{8} h_{9}$. Hence we want to make those monomials dominant. To achieve this we use the following transformation (based on the normal vector of the hyperplane):

34 $$\begin{aligned} h_{1}\rightarrow t,\quad h_{2}\rightarrow t,\quad h_{3}\rightarrow t,\quad h_{6}\rightarrow t. \end{aligned}$$

The result is the following degree 18 polynomial in *t*, with coefficients that are polynomials in $h_4$, $h_5$ and $h_7$, ..., $h_{10}$:

$$\begin{aligned} \begin{aligned} D_6(t)&= 324 t^{18} (h_{10}+h_{7}) (h_{10} h_{7}-h_{8} h_{9}) + \cdots \\&\quad + h_{10} h_{4} h_{5} h_{7} h_{8} h_{9} \\&\quad \cdot (h_{10}+h_{4}) (h_{10}+h_{5}) (h_{10}+h_{7}) (h_{10}+h_{8}) (h_{10}+h_{9}) \\&\quad \cdot (h_{4}+h_{5}) (h_{4}+h_{7}) (h_{4}+h_{8}) (h_{4}+h_{9}) \\&\quad \cdot (h_{5}+h_{7}) (h_{5}+h_{8}) (h_{5}+h_{9}) \\&\quad \cdot (h_{7}+h_{8}) (h_{7}+h_{9}) \\&\quad \cdot (h_{8}+h_{9}). \end{aligned} \end{aligned}$$

As the constant coefficient is a sum of positive monomials one has $D_6(0)>0$. Thus, if

$$\begin{aligned} h_{10} h_{7}-h_{8} h_{9} < 0, \end{aligned}$$

then there exists a $t_1>0$ such that $D_6(t_1)=0$ and $D_6 (t)<0$ for $t>t_1$ by the Intermediate Value Theorem. These observations are the basis for the following result:

#### Lemma 3.3

Consider the coefficients of the characteristic polynomial given in (31) for $\lambda =1$ and obtain its coefficients $b_1(h), \ldots ,$ $b_7(h)$ and its Hurwitz-matrices $G_1(h)$, ..., $G_6(h)$. Let *h*(*t*) be such that

35 $$\begin{aligned} h(t) = \left( t, t, t, h_4, h_5, t, h_7, h_8, h_9, h_{10} \right) ^T \end{aligned}$$

with

36 $$\begin{aligned} h_{7}h_{10}-h_{8} h_{9} < 0. \end{aligned}$$

Let $b_i(h(t))= b_i (t)$ and $D_i (t) =\det G_i (h(t))$, $i=1$, ..., 6. Then there exists a positive real number $t_1$, such that

$$\begin{aligned} D_6(t) < 0 \text { for } t >t_1 \quad \textrm{and}\quad D_6(t_1) = 0. \end{aligned}$$

In addition, $b_7(t_1)>0$ and $D_i (t_1) >0$ for $i=1,2, \ldots , 5$.

#### Proof

Choose positive values $h_4^*$, $h_5^*$ and positive values $h_7^*$, $h_8^*$, $h_9^*$ and $h_{10}^*$ that satisfy (36). Fix $h_4=h_4^*$, $h_5=h_5^*$ and $h_7=h_7^*$, ..., $h_{10} = h_{10}^*$ to obtain $h^*(t)$ (which now depends only on *t*). Evaluate the $b_i$’s and $\det (G_i)$ at $h^*(t)$ to obtain the *t*-polynomials $b_i(t)\equiv b_i(h^*(t))$ and $D_i(t)\equiv \det (G_i(h^*(t)))$.

The existence of $t_1$ with $D_6(t)<0$, $t>t_1$ and $D_6(t_1)=0$ has been established above. By Lemma 3.2$b_1$, ...$b_5$ and $b_7$ as well as $\det (G_2)$, ..., $\det (G_5)$ are sums of positive monomials and thus, in particular, positive if evaluated at $h^*(t_1)$. Thus $b_7(t_1)$ and $D_i(t_1)$, $i=1,2, \ldots ,5$ are positive. $\square$

#### Theorem 3.4

Consider the dynamical system (25a)–(25j) arising from network (3) with Jacobian $J_{\lambda }(h)$ as in (30). Let *h*(*t*) be as in (35) and $\lambda >0$. Fix the remaining $h_i=h_i^*$ such that the inequality (36) is satisfied. Then for $t=t_1$ computed as in Lemma 3.3, the dynamical system (25a)–(25j) undergoes a simple Hopf bifurcation for all $\lambda >0$ if

$$\begin{aligned} \frac{d \det (G_6(t))}{d t}\Bigg |_{t=t_1} \ne 0. \end{aligned}$$

#### Proof

If $t=t_1$ is as in Lemma 3.3, the conditions (32) of Corollary 3.1 are satisfied. If $\frac{d \det (G_6)}{d t}|_{t=t_1} \ne 0$, then at least one of the derivatives $\frac{\partial \det (G_6)}{\partial h_i}|_{h=h(t_1)} \ne 0$ (by the chain rule) and hence condition (33) is satisfied. Thus, by Corollary 3.1, the dynamical system (1) undergoes a Hopf bifurcation at $t=t_1$ in this case. $\square$

Theorem 3.4 establishes the existence of simple Hopf bifurcations for the the dynamical system (25a)–(25j) derived from network (3). In the following remark we observe that inequality (2) and inequality (36) are equivalent:

#### Remark 3.5

First recall that the $h_i$ have to satisfy (29) [as ($\kappa$,$\frac{1}{h}$) is a solution to the steady state Eq. (10)]. That is, $h_7$, ..., $h_{10}$ can be represented in terms of $\kappa _3$, $\kappa _6$, $\kappa _9$ and $\kappa _{12}$ (and $\lambda$) as

$$\begin{aligned} h_{7} = \frac{\kappa _3}{\lambda },\quad h_{8} = \frac{\kappa _6}{\lambda },\quad h_{9} = \frac{\kappa _{12}}{\lambda },\quad h_{10} = \frac{\kappa _9}{\lambda }. \end{aligned}$$

Using this one obtains for the left hand side of inequality (36):

$$\begin{aligned} h_7 h_{10} - h_8 h_9 = \frac{1}{\lambda ^2}\left( \kappa _3 \kappa _9 - \kappa _6 \kappa _{12} \right) . \end{aligned}$$

As $\lambda ^2>0$ the inequality (36) is equivalent

$$\begin{aligned} \kappa _3 \kappa _9 - \kappa _6 \kappa _{12} < 0. \end{aligned}$$

Finally, we make several remarks regarding the stability of the positive steady state $x^*(t)=\frac{1}{h(t)}$ of the system (25a)–(25j) depending on $t>0$.

#### Remark 3.6

- For sufficiently small $t>0$, the positive steady state $x^*(t)$ is asymptotically stable. (This is true regardless of inequality (36)).
- Suppose that the inequality (36) is satisfied. Then for sufficiently large $t >0$, $x^*(t)$ is unstable.

### A procedure to locate simple Hopf bifurcations in network (3)

**Fig. 2:**
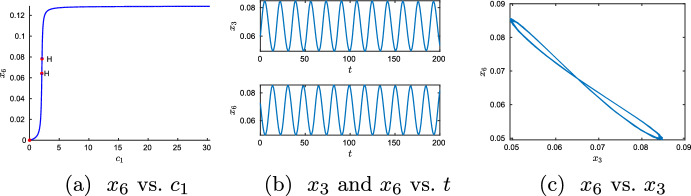
Numerical verification of Hopf bifurcations (**a**, labeled *H*) and a limit cycle (**b**, **c**). Rate constants *k* as in Table 2 with $\lambda =1$. Initial value *x*(0) as in (37)—apart from $x_3(0)$ and $x_6(0)$: to obtain an initial value near the steady state *x* given in (37) we choose $x_3(0) = 1.1\cdot \frac{1}{15}$ and $x_6(0)=0.9\cdot \frac{1}{15}$)

Following the proof of Lemma 3.3 we proceed as follows to find points that satisfy (32):

- Step 1: Choose positive values for $h_{7}$, $h_{8}$, $h_{9}$, $h_{10}$ such that (36) is satisfied (e.g. $h_{7}=h_{8}=h_{10}=1$ and $h_{9}=2$).
- Step 2: Choose positive values for $h_4$ and $h_5$ (e.g. $h_4 = h_5 =1$).
- Step 3: In *p*(*t*) set $h_{1}=h_{2}=h_{3}=h_{6}= t$. For the values chosen so far we obtain
  $$\begin{aligned} \begin{aligned}&497664 + 10946304 t + 103721056 t^2 + 579850652 t^3 + 2169242876 t^4 \\&\quad + 5787611019 t^5 + 11398671182 t^6 + 16865933820 t^7 + 18863357157 t^8 \\&\quad + 15900121640 t^9 + 9989687485 t^{10} + 4589099030 t^{11} + 1497364081 t^{12} \\&\quad + 331280824 t^{13} + 45135703 t^{14} + 2794428 t^{15} \\&\quad - 85122 t^{16} - 20304 t^{17} - 648 t^{18} = 0 \end{aligned} \end{aligned}$$
- Step 4: Approximate the positive real root(s) of $p(t)=0$. For the values chosen so far we obtain $t^*\approx 14.874$.
- Step 5: Check that the point generated so far satisfies (33). In the example we obtain
  $$\begin{aligned} h^*=(14.874,14.874,14.874,1,1,14.874,1,1,2,1)^T. \end{aligned}$$
  We use Matcont to verify a Hopf bifurcation, cf. Fig. 2a.
- Step 6: Choose some $t>t^*$, compute vectors *h* and $x=\frac{1}{h}$ and use Eqs. (14) and (26) to obtain rate constants and total concentrations. We have chosen $t=15$ and hence obtain
  37 $$\begin{aligned} h^T= & {} (15,15,15,1,1,15,1,1,2,1) \quad \textrm{and} \nonumber \\ x^T= & {} \left( \frac{1}{15},\frac{1}{15},\frac{1}{15},1,1,\frac{1}{15},1,1,\frac{1}{2}, 1 \right) \end{aligned}$$
  and the rate constants and total concentrations given in Table 2.

#### Remark 3.7

As a consequence of Lemma 3.3, any point $x^*$ obtained via Steps 1–5 is a *candidate* Hopf point. It is guaranteed to satisfy (32), but a simple Hopf bifurcation only occurs if the condition (33) is satisfied as well. In practice we suggest the following approach: first determine a candidate point $x^*$ via Steps 1 to 5, second use (29) and (26) to determine the corresponding rate constants and total concentrations and third verify the existence of a simple Hopf bifurcation by using a numerical continuation software like MATCONT (Kuznetsov 2003) to vary a rate constant or total concentration at this point.

**Table 2:** Rate constants and total concentrations obtained by solving (13) for *k*

$\kappa _1$	$\kappa _3$	$\kappa _4$	$\kappa _6$	$\kappa _7$	$\kappa _9$	$\kappa _{10}$	$\kappa _{12}$	$c_1$	$c_2$	$c_3$
225 $\lambda$	$\lambda$	15 $\lambda$	$\lambda$	225 $\lambda$	$\lambda$	15 $\lambda$	2 $\lambda$	$\frac{31}{15}$	$\frac{47}{30}$	$\frac{169}{30}$

#### Remark 3.8

In the course of Steps 1–6 above all rate constants are fixed:

(i) From (14) one obtains for network (3) the relation (29) between the $\kappa _i$ and the $h_i$.
(ii) Thus, choosing numerical values for $h_7$, ..., $h_{10}$ is equivalent to choosing $\kappa _3$, $\kappa _6$, $\kappa _9$ and $\kappa _{12}$ (up to the factor $\lambda$).
(iii) Choosing numerical values for $h_4$ and $h_5$ and assigning $h_1=h_2=h_3=h_6=t$ is equivalent to choosing $\kappa _1$, $\kappa _4$, $\kappa _7$ and $\kappa _{10}$ (again up to the factor $\lambda$). In this case $\kappa _1$ and $\kappa _7$ are proportional to $t^2$ and $\kappa _4$ and $\kappa _{10}$ to *t* – as $h_4$ and $h_5$ are fixed to numerical values.
(iv) For $h_7$, ..., $h_{10}$ chosen in Step 1 and $\lambda =1$ one thus obtains $k=\left( t^2, 1, t, 1, t^2, 1, t, 2 \right)$.

### Lifting to the full network (1)

In Banaji (2018) network modifications are described that preserve the existence of a stable positive limit cycle. This is the basis for the following result:

#### Theorem 3.9

Consider networks (1) and (3). Assume the rate constant values of network (3) are such that the system (25a)–(25j) admits a stable limit cycle. Choose these values for the rate constants of network (1). For values of $\kappa _2$, $\kappa _5$, $\kappa _8$, $\kappa _{11}$ small enough, there exists a stable limit cycle in the system (24a)–(24j) close to the limit cycle of the system (25a)–(25j).

#### Proof

In the language of Banaji (2018), if, a network that admits a stable positive limit cycle (for some values of the rate constants and initial conditions) is modified by adding reactions that are in the span of the stoichiometric matrix, then the new network also admits a stable positive limit cycle (Banaji 2018, Theorem 1) (if the rate constants of the new reactions are chosen appropriately).

As the reaction vectors of reversible reactions are in the span of the stoichiometric matrix, the existence of a limit cycle in network (3) implies the existence of a limit cycle in the full network (1) for appropriately chosen rate constants of the backward reactions. $\square$

**Fig. 3:**
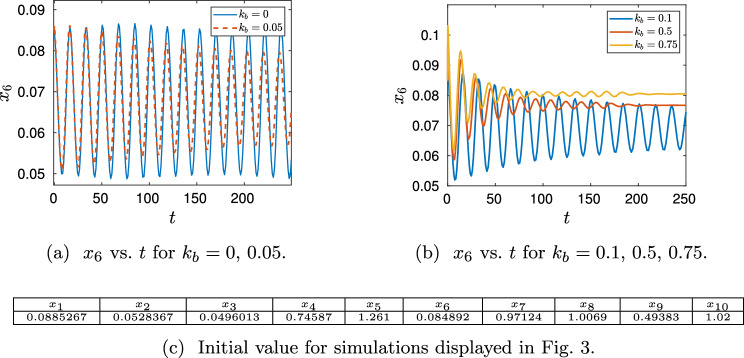
Simulation of network (1) for $\kappa _2 = \kappa _5 = \kappa _8 = \kappa _{11} = k_b$ and different values $k_b$ (ODEs have been solved with ode15s (Mathworks) for *x*(0) as in the table of **c** and $\kappa _i$, $c_i$ as in Table 2 (on p. 16 with $\lambda =1$). **a** $k_b=0$ corresponds to the ODEs derived from network (3), $k_b=0.05$ to the ODES (24a)–(24j) for $k_b=0.05$. The oscillations indicate for $k_b=0.05$ a stable limit cycle close to the stable limit cycle for $k_b=0$. **b** the stable limit cycle does not seem to exist for larger values of $k_b$

To illustrate Theorem 3.9, we use the ODEs (24a)–(24j) and the values of Table 2 (on page 16). Figure 3 demonstrates the existence of a limit cycle in the full system (24a)–(24j)(for $k_b$ sufficiently small).

#### Remark 3.10

(*Locating the limit cycle in Fig.* 3a) For simplicity we choose $\kappa _2 = \kappa _5 = \kappa _8 = \kappa _{11} = k_b$. To obtain Fig. 3 the initial value given in the table of Fig. 3c was used. This point is ‘close’ to the limit cycle of the ODEs defined by network (3) (i.e. for $k_b=0$). It was obtained by solving the ODEs (24a)–(24j) using Matlab’s ode15s with initial value given in Table 2 (on p. 16) for a ‘long’ time (i.e. until $T=5000$) and $\lambda =1$. The point in the table of Fig. 3c corresponds to the last point of that first simulation.

## Discussion

In this section we discuss inequality (2) in the light of results on multistationarity for a network of sequential distributive double phosphorylation described in Conradi and Mincheva (2014). In Sect. 4.1 we introduce the corresponding reaction network and compare it to network (1). In Sect. 4.2 we briefly summarize the results presented in Sect. 3 and in Sect. 4.3 the multistationarity results of Conradi and Mincheva (2014). We close by arguing our conclusion that in distributive double phosphorylation the catalytic constants enable non-trivial dynamics in Sect. 4.4.

### Cyclic versus sequential distributive double phosphorylation

Sequential and distributive double phosphorylation can be described by the following mass action network (cf. e.g. Holstein et al. 2013 or Conradi and Mincheva 2014):38Network (38) is structurally similar to network (1): both networks contain 12 reactions and the only difference is that network (1) contains two species of mono-phosphorylated protein ($S_{10}$ and $S_{01}$), while network(38) contains only one ($S_1$). Hence network (38) contains nine species, while network network (1) contains ten.

In particular, networks (1) and (38) contain the same four phosphorylation events: (i) the conversion of unphosphorylated protein to mono-phosphorylated protein catalyzed be the kinase *K*, (ii) the conversion of mono-phosphorylated protein to double-phosphorylated protein catalyzed by the same kinase *K*, (iii) the conversion of double-phosphorylated protein to mono-phosphorylated protein catalyzed by the phosphatase *F* and (iv) the conversion of mono-phosphorylated protein to unphosphorylated protein catalyzed by the same phosphatase *F*. As described in Conradi and Mincheva (2014), in enzyme kinetics research it is customary to characterize such phosphorylation events by three constants, the Michaelis constant ($K_m$), the catalytic constant ($k_c$) and the equilibrium constant $k_{eq}$ of the respective enzyme substrate pair (see, for example, Bowden (2004) for details on enzyme kinetics).

Of particular interest in the context of the present publication are the $k_c$-values as these correspond to the rate constants involved in inequality (2): $\kappa _3$ is the $k_c$-value of the kinase *K* with unphosphorylated substrate ($S_{00}$ or $S_0$), $\kappa _6$ of *K* with mono-phosphorylated substrate ($S_{10}$ or $S_1$), $\kappa _9$ of *F* with double-phosphorylated substrate ($S_{11}$ or $S_2$) and $\kappa _{12}$ of *F* with mono-phosphorylated substrate ($S_{10}$ or $S_1$).

### Cyclic and distributive: emergence of oscillations

By Theorem 3.4, if these catalytic constants satisfy inequality (2), then there exists positive steady states of network (3) such that the Jacobian has a complex-conjugate pair of eigenvalues on the imaginary axis. This is necessary for a simple Hopf-bifurcation. If there is a supercritical simple Hopf bifurcation and a stable limit cycle emerges, then by Theorem 3.9 there is a stable limit cycle in network (1). Hence we say that for cyclic and distributive double phosphorylation the catalytic constants enable the emergence of oscillations.

### Sequential and distributive: emergence of bistability

In Conradi and Mincheva (2014) we have shown that the inequality (2) is sufficient for multistationarity in network (38). To be more precise, by Conradi and Mincheva (2014, Theorem 5.1), if the catalytic constants satisfy inequality (2), then there exists values of the total concentrations of kinase, phosphatase and protein such that network (38) has three positive steady states – no matter what values the other rate constants take. As multistationarity is necessary for bistability, we say in Conradi and Mincheva (2014), that the catalytic constants enable emergence of bistability in sequential and distributive double phosphorylation.

### Catalytic constants and non-trivial dynamics

In the previous subsections we have described how the catalytic constants of cyclic distributive double phosphorylation enable the emergence of oscillations, and how the catalytic constants of sequential distributive double phosphorylation enable the emergence of bistability. Hence we conclude that in distributive double phosphorylation the catalytic constants enable non-trivial dynamics.

As a consequence, if the rate constant are chosen according to the procedure of Sect. 3.3 and Theorem 3.9 and network (1) admits a stable limit cycle for these rate constants, then network (38) taken with the *same rate constant values* will show multistationarity—for some, usually different, value of the total concentrations. That is, if the catalytic constants satisfy (2), then a *cyclic mechanism* can show *sustained oscillations*, while a *sequential mechanism* equipped with the same rate constant values can show *bistability*.

As an example the procedure described in Sect. 3.3 together with Theorem 3.9 have been used to obtain the following rate constant values:

39 $$\begin{aligned}&\kappa _{1} = 49 \quad \kappa _{2} = \frac{1}{10} \quad \kappa _{3} = \frac{1}{2} \quad \kappa _{4} = 7 \nonumber \\&\kappa _{5} = \frac{1}{10} \quad \kappa _{6} =2 \quad \kappa _{7} =49 \quad \kappa _{8} =\frac{1}{10} \nonumber \\&\kappa _{9} =\frac{1}{4} \quad \kappa _{10} =7 \quad \kappa _{11}=\frac{1}{10} \quad \kappa _{12}=\frac{3}{4} \end{aligned}$$

**Fig. 4:**
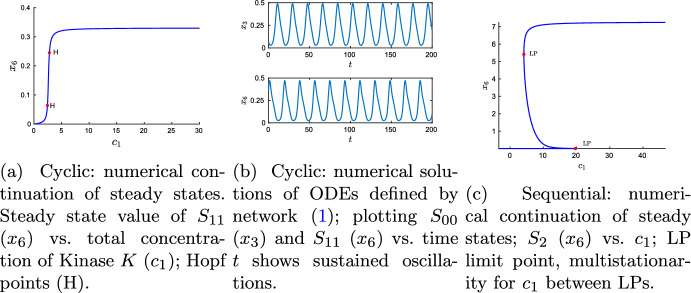
Oscillations and multistationarity in distributive phosphorylation. **a**, **b** Hopf bifurcations (H) and sustained oscillations in network (1); **c** multistationarity in network (38). Rate constants for both networks as in (39), total concentrations for network (1) in Eq. (40) and for network (38) in Eq. (41)

These values satisfy inequality (2). Using these values in the ODEs derived from network (1), one detects simple Hopf bifurcations and oscillations as depicted in Fig. 4a, b. And using these values in the ODEs derived from network (38), one obtains multistationarity as depicted in Fig. 4c. To create these figures, the same parameter values have been used in both ODE systems, albeit for different values of the total concentrations. For Fig. 4a, b the procedure of Sect. 3.3 yields

40 $$\begin{aligned} c_1 = \frac{37}{14},\quad c_2 =\frac{115}{21} \quad \text {and}\quad c_3=\frac{425}{42}, \end{aligned}$$

where $c_1$ denotes total amount of kinase *K*, $c_2$ of phosphatase *F* and $c_3$ of substrate *S*. And for Fig. 4c following the results of Conradi and Mincheva (2014) yields (using the same notation for the total concentrations)

41 $$\begin{aligned} c_1=\frac{307}{27},\quad c_2=\frac{650}{27}\quad \textrm{and}\quad c_3=\frac{18539}{540}. \end{aligned}$$

### Supplementary Information

Below is the link to the electronic supplementary material.

Supplementary file 1 (nb 58 KB)

Supplementary file 2 (txt 155 KB)

## Data Availability

Data sharing not applicable to this article as no datasets were generated or analyzed during the current study.

## A Remark on matrices of the form $A=\lambda B$

Throughout this section let $A, B\in {\mathbb {R}}^{n\times n}$ and $\lambda$ be a nonzero real number such that

$$\begin{aligned} A = \lambda B \end{aligned}$$

### Remark A.1

For matrices *A*, *B* as above one has

because of the multilinearity of the determinant.

As in, for example, Horn and Johnson (2012), for $A\in {\mathbb {R}}^{n\times n}$ let $E_k(A)$ denote the sum of the principal minors of size *k* of the matrix *A*.

### Lemma A.2

For matrices *A*, *B* as above one has

$$\begin{aligned} E_k(A) = \lambda ^k E_k(B). \end{aligned}$$

### Proof

This follows from Remark A.1 and the fact that $E_k(A)$ is a sum of determinants of $k\times k$-sub-matrices. $\square$

Let $a_k$ denote the coefficients of the characteristic polynomial of *A* and $b_k$ those of the characteristic polynomial of *B*.

### Lemma A.3

For *A*, *B* as above

$$\begin{aligned} a_k = \lambda ^k b_k. \end{aligned}$$

### Proof

This follows from Lemma A.2 and and the fact that the coefficients $a_k$ of any $n\times n$ matrix can be defined in terms of the sums of $k\times k$ principal minors of *A* [cf. Horn and Johnson 2012, Eq. (1.2.13)]:

$$\begin{aligned} a_k=(-1)^{n-k} E_k(A). \end{aligned}$$

$$\square $$

And finally:

### Lemma A.4

Let *A*, *B* be as above and assume that ${{\,\textrm{rank}\,}}(A)={{\,\textrm{rank}\,}}(B)=s<n$. Then the characteristic polynomial of *A* can be expressed in terms of the characteristic polynomial of *B* by the following formula:

42 $$\begin{aligned} \det (\mu I_n - \lambda A)&= \mu ^{n-s} \left( \mu ^s + \lambda b_1 \mu ^{s-1} + \cdots + \lambda ^{s-1} b_{s-1} \mu + \lambda ^ s b_s \right) \end{aligned}$$

43 $$\begin{aligned}&= \mu ^{n-s} \lambda ^s \left( \left( \frac{\mu }{\lambda }\right) ^s + \sum _{i=1}^s \lambda ^i b_i(h) \left( \frac{\mu }{\lambda }\right) ^{s-i} \right) \end{aligned}$$

### Proof

Equation (42) follows from Lemma A.2 and Horn and Johnson [2012, Eq. (1.2.13)], Eq. (43) by factoring $\lambda ^s$ (which is well defined as in our setting $\lambda \ne 0$). $\square$

## Hurwitz determinants of $H(\lambda ,h)$ and *G*(*h*)

Recall that $s ={{\,\textrm{rank}\,}}(S)$, where *S* is the $n \times m$ stoichiometric matrix. By Corollary 2.2 we have $a_i =\lambda ^i b_i(h)$, $i=1,2, \ldots , s$.

We use the following formula for the determinant of a matrix *A* with elements $a_{ij}$, *i*, $j=1$, ...*n* proved in Maybee et al. (1989)

44 $$\begin{aligned} \det A = \sum _{\sigma } (-1)^{n+f} A[c_1] \ldots A[c_f], \end{aligned}$$

where $c_1, \ldots , c_f$ are pairwise disjoint cycles of a permutation $\sigma$. For a cycle $c=(i_1, i_2, \ldots ,i_k)$ we have,

$$\begin{aligned} A[c] = a_{i_2i_1} a_{i_3i_1} \ldots a_{i_1,i_k}. \end{aligned}$$

We define the differences between two consecutive indices in a cycle $c=(i_1, \ldots , i_k)$:

### Definition 3

Let $c=(i_1, i_2, \ldots , i_k)$ be a cycle. We define the *(cycle) differences* $d_{i_s i_{s+1}} =i_s -i_{s+1}$, $s=1, \ldots ,k$, where $i_{k+1}=i_1$ between any two consecutive indices of *c*.

The lemma below follows immediately by (44) and the differences’ definition. In (46) we state a different formulation of the product $H_l[c]$, with the help of the differences of a cycle *c*, which is specific to Hurwitz matrices. The same lemma applies to the Hurwitz matrices $G_l (h)$, $l=1,2, \ldots , s$.

### Lemma B.1

Let $\det H_{l}$ be the Hurwitz determinant of *l*-th order, $l=1,2, \ldots , s$. Then

45 $$\begin{aligned} \det H_{l} = \sum _{\sigma } (-1)^{l+f} H_{l} [c_1]\ldots H_{l} [c_f] \end{aligned}$$

where $\sigma$ is a permutation and $c_1, \ldots , c_f$ are the set of corresponding pairwise disjoint cycles of $\sigma$. We have for a cycle $c =(i_1,\ldots , i_k)$ and the corresponding product

46 $$\begin{aligned} H_{l}[c] =a_{2i_1-i_2} a_{2i_2-i_3} \ldots a_{2i_k-i_1} =a_{i_1+d_{i_1 i_2}} a_{i_2+d_{i_2 i_3}} \ldots a_{i_k+d_{i_k i_1}}. \end{aligned}$$

The next lemma follows immediately by Definition 3.

### Lemma B.2

Let $c=(i_1, i_2, \ldots , i_k)$ be a cycle. For any cycle *c*, the sum of its differences is zero, $d_{i_1 i_{2}} +d_{i_2 i_{3}} +\ldots +d_{i_k i_{1}} =0\,.$

### Remark B.3

We notice that $\det H_l (1,h) =\det G_l (h)$ for $l=1,2, \ldots s$ if $\lambda =1$.

Now we can turn to the proof of Proposition 2.4:

### Proof

Let $c=(i_1, \ldots ,i_k)$ be a cycle. By by Corollary 2.2 we have for the product $a_{i_1} \ldots a_{i_k}$

$$\begin{aligned} a_{i_1} \ldots a_{i_k} = \lambda ^{\delta } b_{i_1} \ldots b_{i_k}, \text { where }\delta =i_1 +\ldots + i_k. \end{aligned}$$

By Lemma B.1 and in particular (46)

47 $$\begin{aligned} \begin{aligned} H_l [c]&= a_{i_1 +d_{i_1 i_2}} \ldots a_{i_k +d_{i_k i_1}} = \lambda ^{i_1 +d_{i_1 i_2}} b_{i_1+d_{i_1 i_2}} \ldots \lambda ^{i_k +d_{i_k i_1}} b_{i_k+d_{i_k i_1}} \\&=\lambda ^{\delta } b_{i_1} \ldots b_{i_k} = \lambda ^{\delta } G_l [c] \end{aligned} \end{aligned}$$

where we have used that the sum of the differences of a cycle sum up to zero by Lemma B.2.

Let the sum of the indices of each cycle $c_1,, \ldots c_f$ of a permutation $\sigma$ be denoted by $\delta _1$, ...,$\delta _f$, correspondingly. We use the fact that the cycles $\{c_1, \ldots ,c_f \}$ in a permutation $\sigma$ are pairwise disjoint. Thus it follows that

48 $$\begin{aligned} \delta _1 + \delta _2 + \cdots + \delta _f = 1 +2 + \cdots + l = \frac{l(l+1)}{2}. \end{aligned}$$

By Lemmas B.1, (47) and (48) we have

$$\begin{aligned} \det H_l (\lambda , h)&= \Sigma _{\sigma } (-1)^{l+f} H_l [c_1] \ldots H_l [c_f] \\&= \Sigma _{\sigma } (-1)^{l+f} \lambda ^{\delta _1} G_l [c_1] \ldots \lambda ^{\delta _f} G_l [c_f] \\&= \Sigma _{\sigma } (-1)^{l+f} \lambda ^{\delta _1 +\ldots +\delta _f} G_l [c_1] \ldots G_l [c_f] \\&= \lambda ^{l(l+1)/2} \Sigma _{\sigma } (-1)^{l+f} G_l [c_1] \ldots G_l [c_f]\\&= \lambda ^{l(l+1)/2} \det G_l (h). \end{aligned}$$

Thus, $$\det H_l (\lambda , h) =\lambda ^{l (l+1)/2} \det G_l (h)$$ for $$l=1,2, \ldots , s$$. $$\square $$

## Data for network (3)

Given the species ordering of Table 5, the stoichiometric matrix *S*, the exponent matrix *Y* and a matrix *W* defining the conservation relations:

$$\begin{aligned} \begin{aligned} \tiny S&= \left[ \begin{array}{rrrrrrrr} -1 &{} 1 &{} -1 &{} 1 &{} 0 &{} 0 &{} 0 &{} 0 \\ 0 &{} 0 &{} 0 &{} 0 &{} -1 &{} 1 &{} -1 &{} 1 \\ -1 &{} 0 &{} 0 &{} 0 &{} 0 &{} 0 &{} 0 &{} 1 \\ 0 &{} 1 &{} -1 &{} 0 &{} 0 &{} 0 &{} 0 &{} 0 \\ 0 &{} 0 &{} 0 &{} 0 &{} 0 &{} 1 &{} -1 &{} 0 \\ 0 &{} 0 &{} 0 &{} 1 &{} -1 &{} 0 &{} 0 &{} 0 \\ 1 &{} -1 &{} 0 &{} 0 &{} 0 &{} 0 &{} 0 &{} 0 \\ 0 &{} 0 &{} 1 &{} -1 &{} 0 &{} 0 &{} 0 &{} 0 \\ 0 &{} 0 &{} 0 &{} 0 &{} 0 &{} 0 &{} 1 &{} -1 \\ 0 &{} 0 &{} 0 &{} 0 &{} 1 &{} -1 &{} 0 &{} 0 \\ \end{array} \right] ,\quad Y= \left[ \begin{array}{rrrrrrrr} 1 &{} 0 &{} 1 &{} 0 &{} 0 &{} 0 &{} 0 &{} 0 \\ 0 &{} 0 &{} 0 &{} 0 &{} 1 &{} 0 &{} 1 &{} 0 \\ 1 &{} 0 &{} 0 &{} 0 &{} 0 &{} 0 &{} 0 &{} 0 \\ 0 &{} 0 &{} 1 &{} 0 &{} 0 &{} 0 &{} 0 &{} 0 \\ 0 &{} 0 &{} 0 &{} 0 &{} 0 &{} 0 &{} 1 &{} 0 \\ 0 &{} 0 &{} 0 &{} 0 &{} 1 &{} 0 &{} 0 &{} 0 \\ 0 &{} 1 &{} 0 &{} 0 &{} 0 &{} 0 &{} 0 &{} 0 \\ 0 &{} 0 &{} 0 &{} 1 &{} 0 &{} 0 &{} 0 &{} 0 \\ 0 &{} 0 &{} 0 &{} 0 &{} 0 &{} 0 &{} 0 &{} 1 \\ 0 &{} 0 &{} 0 &{} 0 &{} 0 &{} 1 &{} 0 &{} 0 \\ \end{array} \right] ,\\ W^T&= \begin{bmatrix} 1&{}0&{}0&{}0&{}0&{}0&{}1&{}1&{}0&{}0\\ 0&{}1&{}0&{}0&{}0&{}0&{}0&{}0&{}1&{}1\\ 0&{}0&{}1&{}1&{}1&{}1&{}1&{}1&{}1&{}1\\ \end{bmatrix} \end{aligned} \end{aligned}$$

The vector of rate functions is

49 $$\begin{aligned} v(k,x) = (\kappa _1 x_1 x_3, \kappa _3x_7, \kappa _4x_1x_4, \kappa _6x_8, \kappa _7 x_2 x_6,\kappa _9x_{10},\kappa _{10}x_2x_5,\kappa _{12}x_9)^T. \end{aligned}$$

The diagonal matrix of rate constants

$$\begin{aligned} {{\,\textrm{diag}\,}}(k)= \left[ \begin{array}{cccccccc} k_{1} &{} 0 &{} 0 &{} 0 &{} 0 &{} 0 &{} 0 &{} 0 \\ 0 &{} k_{2} &{} 0 &{} 0 &{} 0 &{} 0 &{} 0 &{} 0 \\ 0 &{} 0 &{} k_{3} &{} 0 &{} 0 &{} 0 &{} 0 &{} 0 \\ 0 &{} 0 &{} 0 &{} k_{4} &{} 0 &{} 0 &{} 0 &{} 0 \\ 0 &{} 0 &{} 0 &{} 0 &{} k_{5} &{} 0 &{} 0 &{} 0 \\ 0 &{} 0 &{} 0 &{} 0 &{} 0 &{} k_{6} &{} 0 &{} 0\\ 0 &{} 0 &{} 0 &{} 0 &{} 0 &{} 0 &{} k_{7} &{} 0\\ 0 &{} 0 &{} 0 &{} 0 &{} 0 &{} 0 &{} 0 &{} k_{8} \\ \end{array} \right] \end{aligned}$$

The vector of monomials of the rate function *v*(*k*, *x*)

$$\begin{aligned} \phi (k,x) = (x_1 x_3, x_7, x_1x_4, x_8, x_2 x_6,x_{10},x_2x_5,x_9)^T \;. \end{aligned}$$

**Table 3:** Complexes of network (1) and (3)

$$S_{00} + K $$	$$ y^{(1)} =(1, 0, 1, 0, 0, 0, 0, 0, 0, 0)^T $$
$$S_{00} K $$	$$ y^{(2)} =( 0, 0, 0, 0, 0, 0, 1, 0, 0, 0)^T $$
$$S_{10} + K $$	$$ y^{(3)} =( 1, 0, 0, 1, 0, 0, 0, 0, 0, 0)^T $$
$$S_{10} K $$	$$ y^{4)} =( 0, 0, 0, 0, 0, 0, 0, 1, 0, 0)^T $$
$$S_{11} + K $$	$$ y^{(5)} =(1,0,0,0,0,1,0,0,0,0)^T $$
$$S_{11} + F $$	$$ y^{(6)} =( 0, 1, 0, 0, 0, 1, 0, 0, 0, 0)^T $$
$$S_{11} F $$	$$ y^{(7)} =( 0, 0, 0, 0, 0, 0, 0, 0, 0, 1)^T $$
$$S_{01} + F $$	$$ y^{(8)} =( 0, 1, 0, 0, 1, 0, 0, 0, 0, 0)^T $$
$$S_{01} F $$	$$ y^{(9)} =( 0, 0, 0, 0, 0, 0, 0, 0, 1, 0)^T $$
$$S_{00} + F $$	$$ y^{(10)} =( 0, 1, 1, 0, 0, 0, 0, 0, 0, 0)^T $$

## Initial data for Fig. 4a, b

To generate Fig. 4a, b the ODEs derived from the full (reversible) reaction network (1) are used (i.e. the ODEs (24a)–(24j)). In both Fig. 4a, b we use the point displayed in Table 4 as initial value.

**Table 4:** Initial value for Fig. 4a, b

$x_1$	$x_2$	$x_3$	$x_4$	$x_5$	$x_6$	$x_7$	$x_8$	$x_9$	$x_{10}$
$\frac{1}{7}$	$\frac{1}{7}$	$\frac{1}{7}$	1	1	$\frac{1}{7}$	2	$\frac{1}{2}$	$\frac{4}{3}$	4

### Remark D.1

The point in Table 4 is a steady state of the irreversible network (3) generated according to our procedure (with $h_7=\frac{1}{2}$, $h_8=2$, $h_9=\frac{3}{4}$, $h_{10}=\frac{1}{4}$, $h_4=h_5=1$ and $t=1$).

We use Matlab’s ode15s to solve the initial value problem defined by the above ODEs with initial value given in Table 4 and the backward constants $\kappa _2=\kappa _5=\kappa _8=\kappa _{11}=\frac{1}{10}$ (and the remaining constants according to our procedure for the irreversible network). These backward constants are ‘small enough’ in the sense of the results of Banaji (2018), that is, that the reversible network (1) has a limit cycle close to the limit cycle of the irreversible network.

However, on the one hand the steady state of Table 4 (of the irreversible network) is ‘far enough’ from a steady state of the reversible network in the following sense: the solution of the reversible system with initial value given in Table 4 approaches a limit cycle of the reversible system (if approximated with ode15s). If the point given in Table 4 was ‘too close’ to a steady state of the reversible system the solution with it as initial value would approach that steady state (if approximated with ode15s).

On the other hand, Fig. 4a is generated with Matcont using the point given in Table 4 as an initial guess of a steady state of the above ODEs (of the reversible network). And this point is close enough to a steady state of the reversible network for Matcont to converge to a steady state.

## Initial data for Fig. 4c

To obtain Fig. 4c we follow Conradi and Mincheva (2014), where a system of nine ODEs is derived from network (38). As in this reference, we use the variables given in Table 5 to denote the species concentrations. As this network does not distinguish $S_{10}$ and $S_{01}$, there is only one mono-phosphorylated from of the substrate ($S_1$) and $S_2$ is used to denote the double-phosphorylated substrate. Consequently, this network contains only nine species. It has, however, 12 reactions and the labeling is consistent with network (1).

**Table 5:** Variables and species for network (38) (as in Conradi and Mincheva 2014)

$x_1$	$x_2$	$x_3$	$x_4$	$x_5$	$x_6$	$x_7$	$x_8$	$x_9$
$$S_0$$	*K*	$$K S_0 $$	$$S_1$$	$$K S_1$$	$$S_2$$	*F*	$$F S_2$$	$$F S_1 $$

Using the approach described in Conradi and Mincheva (2014) we obtain the steady state depicted in Table 6. We use this point as a starting point for the numerical continuation in Matcont.

**Table 6:** Starting point for the numerical continuation in Fig. 4c (a steady state of network (38) for the rate constant values of eq. (39) generated as described in Conradi and Mincheva (2014))

$x_1$	$x_2$	$x_3$	$x_4$	$x_5$	$x_6$	$x_7$	$x_8$	$x_9$
$\frac{1}{10}$	1	$\frac{49}{6}$	$\frac{119}{180}$	$\frac{119}{54}$	$\frac{17}{135}$	1	$\frac{476}{27}$	$\frac{49}{9}$
